# One stage bilateral endoscopic sympathectomy under local anesthesia: Is a valid, and safe procedure for treatment of palmer hyperhidrosis?

**DOI:** 10.4103/0972-9941.62529

**Published:** 2010

**Authors:** Mohamed Salah Awad, Awny Elzeftawy, Salah Mansour, Wael Elshelfa

**Affiliations:** Department of Surgery, Zagazig University Hospital, General Surgery Department, Egypt

**Keywords:** Endoscopic, hyperhidrosis, sympathectomy recurrence

## Abstract

**OBJECTIVE:**

Thoracoscopic sympathetic surgery is currently the best treatment for hyperhidrosis, and the success rate is quite high, but poor emphasis has been given to the type of anaesthesia and its application through either one or two stages of surgery. This study has evaluated the operative and postoperative results of one-stage bilateral thoracoscopic sympathectomy under local anaesthesia.

**MATERIALS AND METHODS:**

From 2003 to 2007, n=14 patients with hyperhidrosis of the upper limbs [4 females and 10 males] with a mean age of 28±2.11 year [range 26-44] were included. They were operated on by means of bilateral ETS under local anaesthesia. The mean follow-up was 1.5 years (range 13-24 months).

**RESULTS:**

No operative mortality was recorded. The mean operating room time for the whole bilateral procedure under was 73. 5±14.5 range [60 -120] min most of the patients were discharged the same day after a chest roentgenogram except, only two patients with gustatory sweating one recurrent sweating in the patient who had previously axillary hyperhidrosis. Also among them two patients (20%) experienced a minimal pneumothorax that required no treatment. Postoperative quality of life and satisfaction were excellent and cost was significantly reduced.

**CONCLUSIONS:**

Bilateral thoracoscopic sympathectomy for palmar hyperhidrosis could be safely and effectively performed in patients refusing GA regarding cost and satisfaction.

## INTRODUCTION

Primary hyperhidrosis is characterized by increased sympathetic activity at the upper thoracic ganglia T2 and T3 with no apparent underlying cause and is currently the most debilitating form of hyperhidrosis observed in 0.5-1% of the population.[1,2] The most usual sites of hyperhidrosis are the hands in 25% of cases, armpit in 20% and both in 55%, while plantar hyperhidrosis counts up to 45% of cases.[1] The degree of sweating may reach the status of clear dripping and be aggravated by stress and anxiety, thus resulting in a psychological, social and professional burden for such patients. Conservative treatment is not always effective and often requires repeated courses of therapy that can be dismantling on the long run.[3] Therefore, the interruption of the upper thoracic sympathetic chain under thoracoscopic guidance has been established as the treatment of choice and has been widely adopted since many years in most thoracic surgical centres worldwide due to its safety and ability to achieve definitive cure in a surprisingly high percentage of cases.[4–5] Several minimally invasive techniques have been described so far with different results in terms of resolution of symptoms, complication and need for redo procedures, but poor emphasis has been given to the type of anaesthesia for this type of surgery.[6] This study has evaluated the immediate and long-term results of one-stage bilateral video-assisted thoracoscopic sympathectomy under local anaesthesia (LA) in a small yet significant series of selected patients to demonstrate its feasibility.

## MATERIALS AND METHODS

From 2003 to 2007, n=14 patients suffering from primary hyperhidrosis underwent in this study; they had palmer and axillary hyperhidrosis.

All patients were subjected to clinical assessment that includes history and examination to prevent inappropriate surgical intervention as in patients with secondary hyperhidrosis.

To avoid perioperative morbidity in patients with major pulmonary pathology causing marked pleural adhesions, preoperative chest X-ray was done to exclude pleural thickening or major lung pathology. Routine and special laboratory tests were done such as thyroid profile and plasma catecholamine in suspicious cases to exclude secondary hyperhidrosis.

Each patient signed an informed consent after having been carefully educated on performing under local anaesthesia and on the possible side effects such as transient compensatory sweating, inter-scapular pain and Horner's syndrome.

**Operative technique:** Position: semi-prone position, 20° anti-Trendlenberg with the arms abducted <90°. Monitoring included arterial and central venous blood pressure, electrocardiogram (ECG) and pulse oximetry. On the other hand, a percutaneous intercostal block was performed at the level of the thoracic incisions for a maximum extension of three spaces by infiltration of mixture (lidocaine Hcl and bupivocaine 0.5%) (10 ml] for each intercostal space, 5 cc subcutaneous and 5 cc deep infiltration at each port site, the maximum dose [30-45 cc] for each patient in 7-20 mg/kg body weight. These patients maintained spontaneous breathing throughout the procedure and supplementary oxygen was administered by means of a facemask as required.

n= 3 ports [5 mm] were placed, respectively, in the fourth intercostal space on mid-axillary line and third intercostal space anterior to mid-axillary line (anterior to the border of the latissimus dorsi muscle), the former being used to introduce a 0° telescope while dissection was performed through the other two ports of the third port site 5 mm incision in the fifth intercostal space in the posterior axillary line.

The first side to be approached was always at the right as preferred by most surgeons. The surgical technique usually consisted of opening the parietal pleura.

The sympathetic chain and its ganglia were identified emerging deep to the yellow pad of fat under a layer of parietal pleura and passing inferiorly across the necks of the ribs.

In palmar hyperhidrosis, the 2nd and 3rd thoracic ganglia were cauterized by monoboler diathermy, but in axillary hyperhidrosis the 4th ganglion is excised in addition.

Electrocautery ablation of accessory branches and Kuntz nerve when present was performed in all cases to prevent relapses.

All patients received sedative medication preoperative [medozolam] and intravenous analgesic agents throughout the procedure [pethedine 100 mg], and not all patients needed postoperative anti-inflammatory analgesia.

A temporary 10 Fr thoracic drain was left in place and connected to mild suction, while the patient's position was changed to approach the other side. No further drain was inserted and discharged the same day after a routine postoperative chest roentgenogram to exclude the presence of residual pneumothorax.

At the end of the operation, the camera was removed gradually while the lung was inflated manually to make sure that the lung was fully expanded. Local anaesthetic infiltration at the port site was done to avoid post-operative chest pain. Intercostal tubes were not used.

Chest X-ray was done in all patients postoperatively to exclude pneumothorax or haemothorax. Operative time and hospital stay for all patients were recorded.

Post-operative observations of hand dryness and warmth, and if any complications, were recorded also.

## RESULTS

No operative mortality was recorded. The average time for completion of the procedure range from 60 to 120 min with a mean time 73.5±14.5 min. No patients were converted into open procedure due to pleural adhesions or intra-operative bleeding. In three patients, very thin adhesions were encountered that could be easily eliminated by electrocautery.

In nine patients, supplementary medication was required to control pain with other injection of diclofenac sodium.

Haemodynamic and gas exchange were well maintained throughout the procedure. Immediate relief of symptoms was obtained in most patients n=10 [71.2%] with an excellent degree of satisfaction manifested by a warm and dry hand [Tables 1, 2]

**Table 1:** Preoperative patient's characters

Preoperative patients characters	Patients
Sex m/f	10/4
Age	
median /range	28±2.11[26-44]
Previous chest operations	no
Previous treatments	10/14
Site of the Hydrosis	3/14
palm only	4/14
Axilla only	7/14
Both palm and axilla	

**Table 2:** Operative and postoperative findings

Mean Operative time [range] minutes.	73. 5±14.5 [60 - 120]
Mortality	no
Residual pneumothorax	Two/14[14.6%]
Segmental atelectasis	no
Pain	
[intraoperative supplement medications]	9/14[65%]
Postoperative non steroidal anti-inflammatory	10/14 [72%]
Postoperative one injection	4/14 [28.6%]
More than one injection	4/14 [28.6%]
Post operative Inter-scapular pain	4[28.6]
Bleeding [volume]	70-100cc
Compensatory sweating	3/14 [21,6%]
Horner syndrome	No
Recurrence	1[7.4]

Late complications included inter-scapular pain (n=4, 28.6%) lasting for a mean time of 9 days (range 4-19) and compensatory sweating (n=3, 21.6%) that resolved spontaneously at 6 months.

Mediate term results of hyperhidrosis after a mean follow up (1 year) [range from 6 to 17 months] was 13/14 (93%) of patients.

One case of recurrence was observed that had previously isolated axillary hyperhydrosis.

A cost analysis revealed a statistically advantage 935 [900-1270] LE [Table 3].

**Table 3:** Degree of satisfaction and cost

Degree of satisfaction	(n=14)
Excellent	10 (71.2%)
Very good	2(14.4%)
Good	2(14.4%)
Cost effect of the operation under local anaesthesia	Egyptian pounds LE
• Operative room medications [local anaesthesia+ Sedative] and instruments	Mean [650] Range[456-786]
• Medical members	190 [100-300]
• Hospital stay and post operative treatment	155 [200-270]
Total [mean/range]	935 [900-1270] LE

## DISCUSSION

Bilateral endoscopic sympathectomy can be performed by different surgical and anaesthesiological techniques, yet the degree of patient's compliance and satisfaction for such a procedure must be taken into account. Giving the patient the chance of achieving both functional and aesthetic results with minimal risk and discomfort together with an excellent postoperative quality of life is the gold standard.[6]

In a study done by Baumgartner *et al* in 2009[7] as a comparative study between the surgical and non-surgical treatment for hyperhidrosis they found that bilateral thoracoscopic sympathectomy (BTS) was effective in curing palmar hyperhidrosis in 100% of patients. The safety and overwhelming efficacy of BTS compared to medical management of severe palmoplantar hyperhidrosis is demonstrated. Rather than being a ‘last resort’, BTS can be confidently recommended as first-line treatment for the typical, severe form of palmoplantar hyperhidrosis.

Thomas *et al*[8] demonstrated that topical aluminium chloride hexahydrate therapy and iontophoresis are simple, safe and inexpensive therapies; however, continuous application is required because results are often short-lived, and they may be insufficient. Systemic agents such as anti-cholinergic drugs are tolerated poorly at the dosages required for efficacy and usually are not an option because of their associated toxicity. While botulinum toxin can be used in treatment-resistant cases, numerous painful injections are required, and effects are limited to a few months. Surgical sympathectomy should be reserved for the most severe cases and should be performed only after all other treatments have failed. Although the safety and reliability of treatments for palmoplantar hyperhidrosis have improved dramatically, side effects and compensatory sweating are still common, being potentially severe problems.

This is an early experience on bilateral synchronous trans-thoracic sympathectomy under local anaesthesia and we used the most widely adopted two-port technique since it enables a quick and effective procedure and a low rate of perioperative and postoperative complications with good aesthetic result as well.[3,5]

The introduction of the first trocar was always preceded by dissection down to pleura with metzenbaum scissors in order to make sure that no adhesions were present. Once the pneumothorax had been created, the second trocar would be introduced

Versus the a study done by Georgios *et al*,[9] with a single port they published that complete relief of symptoms was observed in all patients at the 6-month follow-up; 45% experienced compensatory hyperhidrosis. Single-port thoracoscopic sympathectomy produces excellent medical and cosmetic results in patients with hyperhidrosis and is associated with a short hospital stay and a low risk of complications.

Intercostal nerve block with bupivacaine 0.5% reduced the immediate postoperative pain and analgesic requirements; Also we believe that downsizing trocars to 5 mm significantly diminishes postoperative pain even compared to a single 12 mm trocar, as reported by others this agree Stafeno *et al*.[10] The use of CO_2_ insufflations undoubtedly enhances visualization by displacing the lung and expediting the procedure; however, the report of serious complications has prevented us from using this type of aid.

Performing thoracoscopic sympathectomy on a patient without general anaesthesia did not cause any major problems to the surgeon except for the need of stopping and waiting when the patient would cough or if the patient distressed to support him or to increase the analgesic dose.

Sympathectomy under general anaesthesia and a single-lumen endotracheal intubation is a well-established procedure,[11] yet complications related to general anaesthesia cannot be underestimated. This is why we selected this small group of patients to try this procedure under local anaesthesia.

Monopolar diathermy was used in all patients so as to produce the least passage of electric and thermal energy over the sympathetic trunk, particularly for T2 one of our patients developed recurrence during the course of follow up (1 year). Gossot *et al*[12] published that the recurrence rate after a long follow up period (5 years) is 15%.

The simplicity of video-assisted thoracoscopy allowing visualization and reliability of access to the thoracic sympathetic trunk has turned this procedure into excellent alternative for open thoracic sympathectomy. E.T.S allows better protection of the stellate ganglion, thus the risk of Horner's syndrome is absent and facilitates simple access to T4; also, E.T.S can be done bilaterally in the same setting with minimal complications,[9] as we did in asynchronous bilateral sympathectomy without Horner syndrome.

As previously reported by Garcia and Espania (2008)[13] in all cases via video-assisted thorascopic surgery: one or more ganglia between T2 and T5 are usually resected depending on the area affected by hyperhidrosis: T2 for craniofacial hyperhidrosis, T3 and T4 for palmar hyperhidrosis and T3 to T5 for combined palmar and axillary hyperhidrosis, versus our study we had resected the fourth ganglion for patients with axillary hyperhydrosis but it agree the study done by Weksler *et al*.[14] They recommend intervention on the T2 ganglia for facial hyperhidrosis and rubor, on the T3 ganglia for palmar hyperhidrosis and on the T3 and T4 ganglia for axillary hyperhidrosis

Lau *et al*,[15] underwent TES without significant complications. The hospital stay was less than 1 day for all patients except one, who stayed 4 days. Estimated operative blood lost was less than 100 ml and no blood transfusions were required. No Horner's syndrome was suffered. After a mean follow-up of 7.0 months (range 1.2-15.8 months), none of the patients had recurrent symptoms in the palms, but all reported moderate compensatory hyperhydrosis located mainly in the trunk and lower extremities (two patients).

Versus our study, we had three cases [21%] with compensatory sweating who had preoperatively axillary hyperhydrosis.

In a retrospective study done by Li *et al* (2008)[16] to compare two groups, one with only T3 and the other group with T2-T4 sympathetic treatment, the incidence of severe compensatory sweating was significantly lower after T3 sympathectomy (3% vs. 10%). As for satisfaction rate, group T3 was superior to group T2-4 (96.6% vs. 89.6%). The rate of symptom resolution was 100%, and no recurrence was found in either group.

Compensatory sweating remains the most common, and most disabling complication of video-assisted thoracoscopic sympathectomy; we had three cases with compensatory sweating; the efficacy of main trunk cutting or T3 ganglion versus T2-T4 should be studied thoroughly to assess their efficacy in reducing the complication of compensatory sweating.

Rieger *et al*,[17] advised cutting the sympathetic chain for excellent results from T2 to T4-5 is safe and effective and leads in almost 100% of cases to the elimination of palmar and axillary hyperhidrosis.

## CONCLUSION

Bilateral synchronous E.T.S under local anaesthesia constitutes a valid, safe, easy to perform, effective and Away from hazards of general anaesthesia but we need other studies with more number of patients to support this hypothesis.
